# Out-of-Season Epidemic of Respiratory Syncytial Virus during the COVID-19 Pandemic: The High Burden of Child Hospitalization in an Academic Hospital in Southern Italy in 2021

**DOI:** 10.3390/children9060848

**Published:** 2022-06-08

**Authors:** Daniela Loconsole, Francesca Centrone, Caterina Rizzo, Désirée Caselli, Azzurra Orlandi, Fabio Cardinale, Cristina Serio, Paola Giordano, Giuseppe Lassandro, Leonardo Milella, Maria Teresa Ficarella, Maria Elisabetta Baldassarre, Nicola Laforgia, Maria Chironna

**Affiliations:** 1 Hygiene Section, Interdisciplinary Department of Medicine, Aldo Moro University, 70124 Bari, Italy; daniela.loconsole@uniba.it (D.L.); francesca.centrone@uniba.it (F.C.); 2 Clinical Pathways and Epidemiology Function Area, Bambino Gesù Children’s Hospital, IRCCS, Piazza Sant’Onofrio, 4, 00165 Rome, Italy; caterina1.rizzo@opbg.net; 3 Pediatric Infectious Diseases Unit, Giovanni XXIII Children Hospital, Azienda Ospedaliero Universitaria Consorziale Policlinico, 70124 Bari, Italy; desiree.caselli@policlinico.ba.it (D.C.); azzurra.orlandi@policlinico.ba.it (A.O.); 4 Department of Pediatrics, Giovanni XXIII Children Hospital, Azienda Ospedaliero Universitaria Consorziale Policlinico, 70124 Bari, Italy; fabio.cardinale@policlinico.ba.it (F.C.); c.serio9@studenti.uniba.it (C.S.); 5 Pediatric Unit, Interdisciplinary Department of Medicine, Aldo Moro University, 70124 Bari, Italy; paola.giordano@uniba.it (P.G.); giuseppe.lassandro@policlinico.ba.it (G.L.); 6 Pediatric Intensive Care Unit, Giovanni XXIII Children Hospital, Azienda Ospedaliero Universitaria Consorziale Policlinico, 70124 Bari, Italy; leonardo.milella@policlinico.ba.it (L.M.); mariateresa.ficarella@policlinico.ba.it (M.T.F.); 7 Unit of Neonatology and Intensive Care, Department of Biomedical Sciences and Human Oncology, Aldo Moro University, 70124 Bari, Italy; mariaelisabetta.baldassarre@uniba.it; 8 Unit of Neonatology and Intensive Care, Interdisciplinary Department of Medicine, Aldo Moro University, 70124 Bari, Italy; nicola.laforgia@uniba.it

**Keywords:** respiratory syncytial virus (RSV), bronchiolitis, epidemiology, COVID-19, children, hospitalization, prevention

## Abstract

Respiratory syncytial virus (RSV) infection is the most common cause of hospitalization in young children. In the last 2 years, public health measures aimed at controlling the spread of SARS-CoV-2 have affected the epidemiology and seasonality of RSV worldwide. The aim of this descriptive retrospective observational study was to describe the characteristics of children hospitalized with RSV in an academic tertiary care hospital in Southern Italy in 2021. We also investigate the seasonal trends of RSV from 2017 to 2021. The demographic characteristics, comorbidities, clinical data, and coinfections were retrospectively evaluated. Compared with previous seasons, the 2021 outbreak of RSV was characterized by an increased number of patients, with a delayed peak observed in November. Overall, 179 children, including 128 (71.5%) aged <12 months, were hospitalized for RSV infection between August and December 2021. Ten children (5.6%) were admitted to the intensive care unit (ICU), all aged <5 months. One patient (0.5%) aged <1 month with severe comorbidities died. The severity of symptoms was significantly associated with younger age, underlying chronic disease, and the length of hospital stay (*p* < 0.05 each). History of prematurity was not significantly associated with the presence of coinfections. Because of the high burden of RSV infection and the expected larger RSV epidemics resulting from a greater number of RSV-naïve children, systematic epidemiological and virological surveillance is needed. Appropriate pathways for access to RSV prevention in all infants should also be introduced.

## 1. Introduction

Respiratory syncytial virus (RSV) is the leading cause of respiratory infection in young children [1,2,3]. In particular, RSV infection is the leading cause of both death and hospitalization for respiratory illnesses in children aged <1 year [4,5]. More than 60% of children are infected with RSV during the first year of life and almost all are infected before the age of 2 years [6,7]. It has been estimated that 58,000 children aged <5 years are hospitalized annually due to RSV infection in the United States [6]. Overall, RSV is responsible for 5% of deaths worldwide among children aged <5 years, with most RSV-related deaths occurring in infants during the first year of life [8,9].

The seasonal pattern of RSV infection depends on geographic location and climate. In temperate regions of the Northern Hemisphere, RSV typically peaks in the winter months every year [4,10]. In Italy, the annual outbreak usually occurs from late fall to early spring, peaking in January-February [10,11]. Both subtypes of RSV (A and B) co-circulate during annual outbreaks, although one subtype usually predominates each year. The A subtype seems to correlate with a more severe infection [12].

RSV infection causes a wide range of symptoms including upper and lower respiratory tract infections, the latter including bronchiolitis and pneumonia. Bronchiolitis often occurs after the onset of rhinitis and is associated with cough, dyspnea, and wheezing, especially in neonates and infants [13,14]. In younger children, RSV infection can also be characterized by fever, feeding difficulties, and irritability [13]. The main risk factor for RSV infection and hospitalization is being aged between birth and <1 year just before or during an outbreak. Living with siblings, a low birth weight, exposure to cigarette smoke, and lack of breastfeeding increase the risk of severe infection [15]. Severe outcomes and death occur more frequently in preterm infants and infants with congenital heart disease, chronic lung disease, or immunodeficiency syndromes [9]. The economic burden of RSV infections is notable, especially considering the cost of hospitalizations, admission to intensive care units, and length of stay [16].

At present, no national guidelines have approved specific treatments for RSV infections [17]. Severe forms of bronchiolitis may require oxygen administration, non-invasive or invasive ventilation, and hydration or parenteral nutrition [17]. Because of the lack of effective therapies, a reduction in morbidity and mortality from RSV must rely on preventive measures. Prophylaxis with monthly injections of the monoclonal antibody palivizumab to reduce RSV infection is available for children born at a gestational age ≤35 weeks, for children aged <6 months at the beginning of the RSV season and for children aged <2 years with major risk factors [18,19].

In the last 2 years, public health measures aimed at controlling the spread of SARS-CoV-2, such as stay-at-home orders and social distancing, have altered the epidemiology and seasonality of other respiratory viruses [20,21]. Some studies showed a drastic reduction in the number of hospitalizations and a reduction in the length of hospital stay in children with respiratory diseases [22,23]. Moreover, a drop in the total number of pediatric consultations was reported during the 2020 lockdown both in Italy and in Finland [24,25].

It has been reported that the rate of RSV infection decreased markedly in Europe during the pandemic, decreasing from a peak of 2000–2500 cases per week during previous seasonal outbreaks in 2016–2020 to fewer than 700 cases per week in 2020–2021 [26]. In France, the rate of bronchiolitis-correlated hospitalizations decreased by 82.5% and in Belgium, the number of RSV cases in 2020 decreased by 99% compared with the previous three seasons [27,28,29]. Also in Finland, a rapid decrease in RSV cases was observed in 2020 [25]. A study conducted in Spain during the pandemic period revealed a very low burden of bronchiolitis, mostly caused by RSV, and a delayed peak compared with previous years [22]. In Italy, systematic national surveillance of RSV is lacking, therefore very few epidemiological data are available. Some studies have been performed using samples collected for influenza surveillance based on the definition of influenza-like illnesses [12,30]. These studies showed that RSV infection in Italy had almost completely disappeared in children during the 2020–2021 season [30,31,32]. In particular, the rate of acute bronchiolitis was reported to be 84–95% lower in the 2020–2021 season compared with previous seasons [32,33]. In 2021, however, an interseasonal resurgence of RSV was observed in many countries. The aim of the study was to describe the clinical and epidemiological characteristics of children hospitalized for RSV in an academic tertiary care hospital in Southern Italy during the out-seasonal outbreak in 2021. We also investigated the seasonal trends of hospitalized RSV-positive cases, analyzing data on RSV hospitalizations from 2017 to 2021.

## 2. Materials and Methods

This descriptive observational study evaluated children hospitalized for RSV infection in the Policlinico-Giovanni XXIII Hospital of Bari (Italy), a tertiary care hospital in Southern Italy that covers a population of about 158,716 children and has 208 pediatric beds. All pediatric patients hospitalized with a positive real-time PCR test for RSV between 1 January 2017 and 31 December 2021 were considered for the construction of the epidemic curves. Pediatric patients hospitalized with RSV infection between 1 August 2021 and 31 December 2021 were analyzed in-depth using a retrospective collection of their demographic and clinical characteristics, comorbidities, and coinfections.

Nasopharyngeal swabs and/or aspirates were collected in the ward at the time of hospitalization and sent to the Laboratory of Molecular Epidemiology and Public Health of the Hygiene Unit of Policlinico Hospital. Samples were processed immediately or after storage at −80 °C. Nucleic acids were extracted using the STARMag Universal Cartridge Kit (Seegene, Korea) on the automated Nimbus IV platform. Real-time PCR was performed using the AllplexTM Respiratory Panel Assays (Seegene, Korea) to detect 16 different viruses, including influenza A and B viruses, RSV A and B, adenovirus, enterovirus, parainfluenza viruses 1–4, metapneumovirus, bocavirus, rhinovirus, and three coronaviruses, NL63, 229E, and OC43. Samples were also analyzed using AllplexTM SARS-CoV-2 Assays (Seegene, Korea) to detect SARS-CoV-2.

For analytic purposes, the need for oxygen support was considered a criterion for severe symptoms. Statistical analyses were performed with STATA 12.0 software (StataCorp LLC, College Station, TX 77845-4512, USA). Proportions were compared by chi-squared tests, with *p* values ≤ 0.05 considered statistically significant. A multivariate logistic regression model was used to explore associations between severity of clinical signs and age, sex, comorbidities, coinfections, subtype of RSV, history of prematurity, and length of hospital stay.

All procedures performed in the study were in accordance with the ethical standards of the institutional and national research committees, and the 1964 Helsinki declaration and its later amendments or comparable ethical standards. Ethical approval was obtained from the Institutional Review Board at the Apulian Regional Observatory for Epidemiology (n. 614|04 of 4 January 2022), which waived the requirement for informed consent because all data were deidentified.

## 3. Results

**Figure 1:**
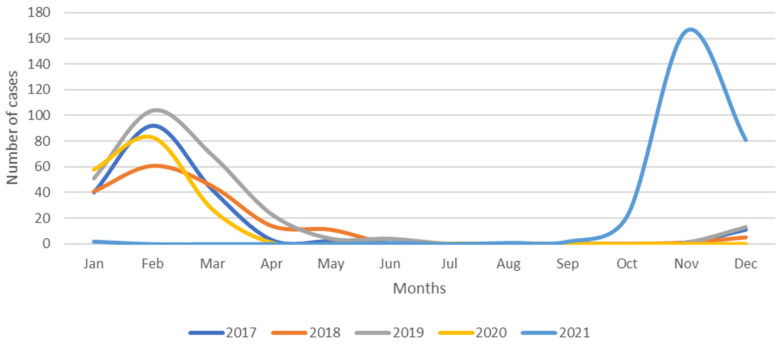
Epidemic curves of RSV hospitalizations in children, years 2017–2021.

Between 1 January 2017 and 31 December 2021, 1082 children were hospitalized for RSV infection. The average number of admissions per year was 216. The 2021 outbreak differed significantly from previous years, as no cases were registered until August 2021 (Figure 1).

Compared with previous seasons, in which hospitalizations peaked during February, the 2021 outbreak began much later in the year and peaked in November. Between August and December 2021, 179 children were hospitalized for RSV infection, including 136 (76%) infected with RSV B, 41 (23%) infected with RSV A, and two (1%) with both. The demographic and clinical characteristics of these patients are summarized in Table 1.

The median age of these 179 children was 4 months (IQR, 1–18 months), with 128 (71.5%) aged <12 months. Eighty-eight children (49.2%) required oxygen support. The average length of hospital stay was 5.8 days (range, 1–16 days). Of the hospitalized children born in 2021, 46.8% were born in September or October. Ten (5.6%) of the 179 hospitalized children were admitted to the intensive care unit (ICU), all of whom were aged <5 months. One patient (0.5%) aged <1 month with severe comorbidities died. Of the 179 children, 67 (37.4%) were coinfected with an additional virus, including six (9%) who were positive for SARS-CoV-2, 32 (47.8%) infected with rhinovirus, 18 (26.9%) infected with bocavirus, 12 (17.9%) infected with adenovirus, and 12 (17.9%) infected with other viruses.

Based on the criteria for the administration of palivizumab, 21 patients were eligible for prophylaxis. Of these, nine children (42.9%) received at least one dose of palivizumab.

Table 2 shows a comparison between the demographic and clinical characteristics of the patients by age group.

Children aged 0–12 months were more likely to show dyspnea (*p* < 0.0001) and cough (*p* = 0.003), whereas older children were more likely to show fever (*p* < 0.0001). Multivariate logistic regression showed that the severity of symptoms was significantly associated with younger age (OR: 0.94, 95% CI, 0.91–0.96), underlying chronic disease (OR: 5.39, 95% CI, 1.7–17.1), and the length of hospital stay (OR: 1.25, 95% CI, 1.1–1.4). Symptom severity was not significantly associated with a history of prematurity, the presence of coinfections, or the subtype of RSV.

## 4. Discussion

The 2021 RSV season did not occur during the expected time of the year, with the present study showing that the first pediatric patient was admitted to hospital for RSV infection in August 2021. Hospitalizations for RSV peaked in November 2021, later than in previous seasons, and were characterized by an increased number of cases. Worldwide, the seasonal pattern of RSV outbreaks was disrupted by the SARS-CoV-2 outbreak and the non-pharmaceutical interventions (NPIs) introduced to contain the spread of the latter virus [34]. During the study period, a low circulation of SARS-CoV-2 was registered in the Apulia region until the beginning of the so-called “fourth wave” in late December 2021 [35]. The average incidence of SARS-CoV-2 infection was 35.9/100,000 inhabitants, ranging from 16.7 to 63.1/100,000 [35]. Social distancing, the use of masks in indoor settings irrespective of vaccination status, and the use of a sanitary passport (vaccination or periodic SARS-CoV-2 testing) were the main public health measures applied to contain the spread of SARS-CoV-2 [36].

In Finland, a rapid decrease in the number of RSV cases in the nationwide lockdown period compared with previous seasons was observed [25]. Also in Spain, during the winter of 2020–2021, a reduction in the rate of RSV diagnoses of 44.3% per thousand inhabitants less than 2 years of age and the rate of RSV admissions of 1.4% per thousand was reported [37]. In addition, a delayed peak of RSV infections was recorded in France during the 2020–2021 season [38]. In particular, the highest number of RSV infections was registered 10–12 weeks after the seasonal peak of previous seasons [38].

Mathematical models have forecast large RSV outbreaks in the next few years due to a buildup of susceptible children [34,39]. In 2021, Australia experienced an interseasonal resurgence of RSV infections with a peak during the austral summer, and the USA also experienced an unusual increase in RSV cases during the spring and summer of 2021 [40,41,42]. Hospital admissions during the Australian interseasonal peak of 2021 included a higher percentage of older children than during previous seasons [40]. In particular, the numbers of hospitalized children aged 12–24 months and 24–48 months were 6.2-fold and 3.4-fold higher, respectively, in 2021 than during previous years [40]. These findings support the hypothesis that higher percentages of older children were RSV-naïve due to SARS-CoV-2 public health measures. Unexpectedly, the present study found that most hospitalized children were aged <12 months, despite the delayed peak of RSV infection. This finding may be due to younger children with the most severe cases being preferentially admitted to a tertiary care hospital.

Most children hospitalized for RSV infection are healthy infants born at term [43]. The present study also found that more than 80% of the hospitalized children were born at term and that a history of prematurity was not associated with a more severe form of infection. Moreover, despite underlying chronic conditions being associated with the severity of symptoms, only 20% of the children in this study showed comorbidities. The month of birth has also been reported to be a risk factor for RSV infection [44]. Similarly, this study found that about 50% of the hospitalized children born in 2021 were born in September or October, at the beginning of the RSV epidemic. The length of hospital stay in the present study was nearly 6 days, longer than the 4.4 days reported in Spain and the 2.1 days reported in Australia [40,45]. The extended length of stay in the present study may be due to the younger age of the children hospitalized in Italy.

The present study also found that younger children were more likely to show dyspnea and cough, whereas older children were more likely to show fever. Overall, 5.6% of patients were admitted to the pediatric ICU, a percentage similar to that reported in Spain in 2018 [44]. In the latter study, being aged <2 months and having other underlying chronic conditions were the main risk factors associated with admission to the ICU [44]. In comparison, all patients in the present study admitted to the ICU were aged <5 months and the majority had comorbidities. The death rate among hospitalized children in the present study was 0.5%, similar to the 0.3% reported in Spain [44].

During the COVID-19 pandemic in the 2020–2021 season, rhinovirus was reported to be primarily responsible for acute respiratory syndrome and hospitalizations for bronchiolitis [31,33,45]. This non-enveloped virus is less affected by NPIs, such as handwashing and the use of ethanol-containing disinfectants [46,47]. Interestingly, the present study found that 47.8% of the reported coinfections were caused by rhinovirus, confirming its high circulation during the COVID-19 pandemic.

The community burden of RSV infection in Italy remains underestimated as a systematic surveillance system for RSV infection has not yet been introduced. Clinical manifestations of RSV in children aged <5 years may persist for 14 days after onset, suggesting the need to consider the persistence of these manifestations when evaluating the clinical and socio-economic burden of RSV infections in young children in primary care [48]. Only 3% of RSV-infected children in primary care showed comorbidity, and 5% were born prematurely, suggesting that the majority of infected children were otherwise healthy [48]. Determining the estimated direct and indirect costs of RSV infections should lead to an in-depth evaluation of the possible benefits of extending antibody prophylaxis to all children. Fewer than 50% of eligible children in the present study received RSV prophylaxis, suggesting the need for wider access to palivizumab. However, the unexpected out-of-season outbreak of RSV in 2021 could have affected the administration of palivizumab in the correct time frame. The high burden reported for RSV infections suggests that targeting all children for preventive measures would be a more effective strategy [16]. Moreover, RSV circulation should be monitored in the coming months to better manage the infections and the prophylaxis.

To date, no specific vaccines have been approved to prevent RSV, although their development is among the priorities of the World Health Organization [49]. Two approaches have been suggested for immunization against RSV: maternal immunization for children aged <6 months and pediatric vaccines for children aged >6 months. Maternal immunization during the third trimester of pregnancy could increase the amount of anti-RSV antibodies passed from the mother to the infant. The passive immunization of infants using this strategy is advisable, as any RSV vaccine in infants would not protect them from infection during the first weeks of life when the risk of hospitalization for RSV is higher [11,41].

## 5. Conclusions

Although the RSV 2021 season was delayed in many countries, Southern Italy experienced an out-of-season RSV epidemic, beginning in August and peaking in November 2021. Because of public health measures imposed to prevent the further spread of SARS-CoV-2, larger RSV epidemics are expected over the next few years due to an increase in RSV-naïve children. The community burden of RSV infection is still unclear. A systematic national and regional epidemiological and virological surveillance system is needed in Italy. Moreover, to limit the clinical and socio-economic burdens, methods are needed to provide all infants with access to anti-RSV antibodies.

## Figures and Tables

**Table 1:** Demographic and clinical data of children hospitalized for RSV infection in 2021.

	N	%
**Total**	179	
**Age groups (months)**		
0–12	128	71.5%
13–24	15	8.4%
>24	36	20.1%
**Sex**		
Male	94	52.5%
Female	85	47.5%
**Symptoms**		
Fever	89	49.7%
Dyspnea	133	74.3%
Wheezing	60	33.5%
Cough	155	86.6%
Rhinitis	103	57.5%
Feeding difficulties	83	46.4%
**Comorbidity**	38	21.2%
**Prematurity at birth**	32	17.9%
**Days from symptom onset to hospitalization average (range)**	4.2 (0–45)	
**Length of hospital stay (days) average (range)**	5.8 (1–16)	

**Table 2:** Comparison of demographic and clinical data of children hospitalized for RSV infection by age group.

	Age Groups			*p*-Value
	0–12 Months N (%)	13–24 Months N (%)	>24 Months N (%)	
**Total**	128	15	36	
**Sex**				
Male	64 (50.0%)	9 (60.0%)	21 (58.3%)	0.56
Female	64 (50.0%)	6 (40.0%)	15 (41.7%)	
**Symptoms**				
Fever	49 (38.3%)	11 (73.3%)	29 (80.6%)	<0.00001
Dyspnea	112 (87.5%)	6 (40.0%)	15 (41.7%)	<0.00001
Wheezing	42 (32.8%)	7 (46.7%)	11 (30.6%)	0.51
Cough	117 (91.4%)	13 (86.7%)	25 (69.4%)	0.003
Rhinitis	79 (61.7%)	7 (46.7%)	17 (47.2%)	0.20
Feeding difficulties	63 (49.2%)	6 (40.0%)	14 (38.9%)	0.48
**Comorbidity**	22 (17.2%)	3 (20.0%)	13 (36.1%)	0.049
**Prematurity at birth**	25 (19.5%)	2 (15.4%)	5 (14.3%)	0.66

## Data Availability

Data are available on request from the corresponding author.
